# Motivation and identity among European and Brazilian elite student-athletes: a Bayesian multilevel regression and poststratification analysis

**DOI:** 10.3389/fspor.2025.1560707

**Published:** 2025-04-10

**Authors:** Carlos E. Gonçalves, Humberto M. Carvalho, Ana Fernandes, Serge Éloi, Ricardo T. Quinaud, Luis M. Rama

**Affiliations:** ^1^ University of Coimbra, Faculty of Sport Sciences and Physical Education, Coimbra, Portugal; ^2^ School of Sports, Federal University of Santa Catarina, Florianópolis, Santa Catarina, Brazil; ^3^ LIRTES, University Paris-Est Creteil, Creteil, France; ^4^ Department of Physical Education, University of the Extreme South of Santa Catarina, Criciúma, Santa Catarina, Brazil; ^5^ CIPER, University of Lisbon, Faculty of Human Kinetics, Cruz Quebrada, Dafundo, Portugal

**Keywords:** cross-cultural comparison, universities, sports, multilevel analysis, Bayesian approach

## Abstract

**Introduction:**

The global dimension of elite sport enrolls a vast number of student-athletes and universities around the world. However, the majority of higher education institutions do not share the same vision and policies about education and elite sport. Hence, comparative studies are needed. The goal of the present study was to use Multilevel Regression with Poststratification to estimate variation between Brazilian and European university student-athletes’ motivation toward sports and academics, and two dimensions of identity, affectivity and social identity.

**Methods:**

508 student-athletes (311 Brazilian, competing at the national university games; 197 European, competing at the European University Games) participated in the study and answered to two questionnaires used in cross-cultural research: SAMSAQ, for academic and sport motivation, and BIMS, for social identity and emotions.

**Results:**

For all the variables our estimations showed significant differences between Brazilian and European athletes, with the former expressing higher scores in all dimensions of motivation and identity.

**Discussion:**

The findings suggest that the macro-effect of the organizational and cultural context is the most important source of influence on athletes’ motivation and identity, which appears to be more significant in Brazil. Cultural nuances among European countries and Brazilian states seem to have little impact on the athletes’ responses. It is necessary to move on from an ethnographic stance and assume methodological sophistication as a way to assimilate a body of knowledge that can be subject of comparison and interpretation. Educators need to compare and see what works at a global level, because thousands of elite athletes are enrolled in higher education and feed national and international competitions with their commitment and quality. Our findings highlight the need for policymakers and educational institutions to develop tailored support systems that acknowledge the cultural and organizational differences impacting student-athlete motivation and identity. Implementing flexible academic programs, fostering supportive athletic environments, and promoting dual career pathways are crucial for optimizing the student-athlete experience globally.

## 1 Introduction

The enrollment of elite athletes in higher education is a widespread phenomenon, with numerous individuals representing their universities and clubs in national and international competitions. This global trend is strengthened by policies and funding initiatives from both national and supranational bodies, which directly impact student-athletes through financial support and mobility programs. Prior studies suggest that elite sport can serve as a vehicle for personal development, facilitating the acquisition of psychosocial skills and life competencies (1, 2). Consequently, collaboration between educational institutions and sports organizations is essential for recognizing the skills that athletes develop through both formal education and non-formal athletic experiences (3).

While the collegiate athletics system in the United States is often highlighted for its integration of elite sport and higher education, it is crucial to recognize the distinct characteristics of the Brazilian and European higher education systems. The European Union’s Erasmus program has significantly increased international student-athlete mobility, highlighting a growing global trend (4). This mobility is supported by EU policies aimed at enhancing dual-career opportunities through scholarships and specialized programs (5). However, the European landscape remains diverse, with countries like Portugal or France having comprehensive school sport legislation and higher education institutions offering structured support through government-guided policies (6).

In contrast, Brazil adopts a more liberal, laissez-faire approach to university sports. While the number of student-athletes engaging in international mobility is increasing, as seen globally, the level of institutional support within Brazilian universities varies significantly compared to European models. This disparity underscores the need for comparative studies that transcend ethnocentric limitations and embrace methodological sophistication (7). Such studies are crucial for understanding the diverse experiences of student-athletes and for developing effective policies that support their dual-career pathways.

It is important to acknowledge that the number of student-athletes involved in dual-career pathways within Brazil and Europe is substantial, although precise figures are challenging to obtain due to varying data collection methods and definitions. However, participation in university sports is growing, driven by increasing recognition of the benefits of dual-career development.

Despite these structural disparities, sport is generally viewed as a socially positive force, enhancing institutional reputation, fostering student engagement, and contributing to academic achievement and career readiness. As a result, policymakers and educational stakeholders require empirical data to inform policies and practices that optimize the dual-career experience for student-athletes. Given the heterogeneity in institutional and cultural settings, comparative studies are necessary to elucidate the interactions between academic and athletic identities in various national contexts. Research on student-athletes often centers on specific case studies or localized contexts (8, 9), with few studies offering a broader comparative perspective that includes student-athletes’ own experiences (10). Moreover, the diversity of analytical approaches within sports research, some of which may not adequately address the complexity of the data, underscores the need for methodological rigor. As the field of sport sciences advances toward an internationally integrated framework, it is essential to transcend ethnocentric limitations and adopt robust methodologies that allow for meaningful cross-cultural comparisons.

Although the Brazilian higher education system is structurally similar to the pre-Bologna European model, Brazil’s unique social, geographical, and cultural landscape provides an opportunity for a nuanced comparative analysis. This requires a rigorous approach to measurement and validation, particularly when exploring psychological constructs across diverse settings (11). This commitment to methodological rigor is essential for understanding how constructs like motivation and identity manifest in the complex interplay of academic and athletic pursuits within distinct cultural contexts. Therefore, leveraging robust analytical frameworks that can accommodate the nuances of hierarchical data is crucial for generating accurate and generalizable insights. By employing such sophisticated methodologies, researchers can move beyond localized perspectives and contribute to a deeper, more comprehensive understanding of the global student-athlete experience.This study addresses the following research questions: (i) how do sports and academic motivation, along with affective and social identity, vary among Brazilian and European student-athletes?; (ii) what are the regional differences in these constructs within Europe and across Brazilian sub-regions?

To address these complexities, this study focuses on two key psychological constructs: identity (the extent to which student-athletes identify as students, athletes, or both) and motivation within both domains. These constructs are assessed through the Baller Identity Measurement Scale (BIMS) and the Student-Athletes’ Motivation toward Sports and Academics Questionnaire (SAMSAQ), which have been validated in various cultural contexts, including Brazil and Europe (12–14). Given the hierarchical nature of the data concerning student-athletes’ identity and motivation, this study employs Multilevel Regression with Poststratification (MRP), a robust analytical framework that enables accurate interpretation of hierarchical data structures (15, 16). MRP is particularly useful for our study as it allows us to model individual outcomes as a function of individual and contextual covariates, accounting for the nested data structure. (e.g., students within universities, universities within regions), reducing bias in small-group estimates, and enabling more accurate population-level predictions (17). This is particularly valuable in cross-cultural comparative studies where sample sizes and representativeness may vary across Brazilian and European regions.

Using MRP, this study aims to estimate variation in sports and academic motivation, along with affective and social identity, among Brazilian and European student-athletes. Based on existing literature, we anticipated that regional differences would be observable within Europe and across Brazilian sub-regions, though specific comparative outcomes were not hypothesized.

## 2 Materials and methods

### 2.1 Study design and participants

This study employed an exploratory, cross-sectional design, with no specific hypotheses due to the novel, comparative nature of the topic. The sample comprised 508 student-athletes, including 311 Brazilian athletes competing in national university games and 197 European athletes competing in the European University Games. Data collection occurred through an online survey administered via Google Forms during the Brazilian championships and the 4th European University Sports Association (EUSA) Games. Participation was voluntary and anonymous, with survey administration approved by the Brazilian University Sports Confederation and the EUSA Organizing Committee. All participants provided informed consent, and European participants confirmed their fluency in English.

The sample was further categorized by sport type (individual and team sports), by gender, and by region within Brazil and Europe. Brazilian participants were grouped into five regions based on standards from the Brazilian Institute for Geography and Statistics (IBGE): North, Central-West, South, Northeast, and Southeast. This regional approach accounts for Brazil’s vast geographical, cultural, and socio-economic diversity, which is essential for an accurate comparative analysis. European participants were grouped into five geographical regions (North, South, West, Central, and East) according to the EuroVoc thesaurus classification for geographical groups (https://eurlex.europa.eu/browse/eurovoc.html?params=72#arrow_7206). This classification reflects established geopolitical and cultural divisions within Europe, providing a structured framework for examining potential regional variations in student-athlete experiences.

Potential biases include self-selection bias, as participation was voluntary. Data collected during university games might have introduced a performance bias, as athletes’ responses could be influenced by their immediate competitive experiences. To mitigate these biases, we ensured anonymity and emphasized the importance of honest responses. However, future studies should consider employing diverse data collection methods and incorporating longitudinal designs to minimize these potential limitations.

### 2.2 Instruments

Participants completed validated versions of SAMSAQ (13, 18) and BIMS (14, 19), both administered using a 6-point Likert scale, with responses ranging from 1 (strongly disagree) to 6 (strongly agree). The SAMSAQ measures motivation across three dimensions: (i) Academic motivation, which captures the drive to succeed academically alongside athletic commitments. Example item: “I am willing to put the time to earn excellent grades in my courses.” (ii) Sport motivation, which assesses the desire to achieve athletic success. Example item: “It is worth the effort to be an exceptional athlete in my sport.” (iii) Career Motivation: Reflects motivation to engage in the athletic career. Example item: “I choose to play my sport, because it is something I am interested as career. want to succeed equally in both my sport and my studies.” The SAMSAQ has been validated in both Europe and Brazil, confirming the robustness of its three-factor structure across these diverse cultural contexts.

The BIMS assesses identity across two dimensions: (i) Social Identity: Evaluates the extent to which participants see themselves as student-athletes within a social context. Example item: “I consider myself a sydent athlete.” (ii) Affectivity: Measures emotional investment in one’s athletic role, both positive and negative. Example item for positive affectivity: “When I am a student-athlete, I feel good about myself.” Example item for negative affectivity: “I feel bad about myself when I do poorly when I am not a student-athlete.”

Both questionnaires have undergone validation studies for European (18) and Brazilian contexts (12–14), ensuring cultural relevance and psychometric reliability in assessing motivation and identity factors for student-athletes in these regions. In particular, The Portuguese version of the BIMS (12) has a two-factor structure, Affectivity (α=0.92) and Social Identity (α=0.73), and a confirmatory factor analysis indicated acceptable fit indices (TLI = 0.91, CFI = 0.96, GFI = 0.95). The Portuguese version of the SAMSAQ (12) has a three-factor structure: Academic Motivation (α=0.92), Sport Motivation (α=0.88), and Career Motivation (α=0.85), and a confirmatory factor analysis showed acceptable fit indices (TLI = 0.90, CFI = 0.96, GFI = 0.87), with all factor loading exceeding 0.70.

### 2.3 Data analysis

We estimated individual responses based on individual (gender and sport) and geographic (region) characteristics, using partial pooling for each individual i, indexed by g, s, and r for gender, sport, and region, respectively. This hierarchical approach allows us to model both individual-level (demographic and sport) and regional influences on student-athletes’ identity and motivation across Brazil and Europe. The model for each outcome variable (e.g., social identity, academic motivation) can be expressed as follows:

$$y_{i}∼\text{Norm}(\mu_{i},\sigma )$$

where $y_{i}$ is the response (e.g., social identity, motivation) for individual i. For computational convenience, we assumed that the response is truncated to the interval [1,6].

$$\mu_{i}=\beta_{0}+\gamma_{g}[g_{i}]+\gamma_{s}[s_{i}]+\gamma_{r}[r_{i}]$$

where:

• $\beta_{0}$ is the overall intercept (i.e., the baseline level for the outcome),
• $\gamma_{g}[g_{i}]$, $\gamma_{s}[s_{i}]$, and $\gamma_{r}[r_{i}]$ represent random intercepts for the effects of gender, sport, and region on the response, respectively, modeled as normally distributed random effects:

$$\begin{array}{rl} \gamma_{g}[g_{i}] & ∼N(0,\sigma_{g})\\ \gamma_{s}[s_{i}] & ∼N(0,\sigma_{s})\\ \gamma_{r}[r_{i}] & ∼N(0,\sigma_{r}) \end{array}$$

where $\sigma_{g}$, $\sigma_{s}$, and $\sigma_{r}$ are the standard deviations of the random effects for gender, sport, and region.

We applied weakly informative priors to regularize the model estimates:

$$\begin{array}{rl} \beta_{0} & ∼N(3.5,1.25)\\ \sigma_{g} & ∼N(0,1)\\ \sigma_{s} & ∼N(0,1)\\ \sigma_{r} & ∼N(0,1) \end{array}$$

To estimate outcomes for identity and motivation across specific demographic and geographic groups, we applied MRP. We used data from the Brazilian University Games 2018 and the European University Games 2018 to weight the predicted scores by population frequencies in each poststratification cell c. Each cell’s prediction $\theta_{c}$ was weighted by its frequency $w_{c}$:

$$\theta_{r}=\frac{1}{N_{r}}\sum_{c=1}^{C_{r}}\theta_{c}\cdot w_{c}$$

where $N_{r}$ is the total weight across cells in region r, and $\theta_{c}$ represents the estimated response in cell c.

The multilevel models were fit using a Bayesian framework with the “brms” package (20) in R [R2018], which enables flexibility in Bayesian modeling. Four chains ran in parallel with 500 iterations, including 250 warm-up iterations, for reliable convergence.

## 3 Results

Convergence was assessed using $\hat{R}$ values, which were close to 1 across parameters, indicating good mixing of the chains. Posterior predictive checks (21) showed that the model adequately captured the observed data, suggesting a reasonable fit given our data and assumptions.

The multilevel regression parameters for the BIMS dimensions and the SAMSAQ dimensions are summarised in Tables 1, 2, respectively.

**Table 1:** Posterior estimates and 68% uncertainty intervals for BIMS dimensions, considering gender, sport, and region.

	Social identity	Affectivity
Population-level parameters		
$\beta_{0}$	3.59 (3.04, 4.15)	4.27 (3.22, 5.22)
Group-level parameters		
$\sigma_{\mathrm{gender}}$	0.25 (0.06, 0.74)	4.33 (3.42, 5.25)
$\sigma_{\mathrm{sport}}$	0.48 (0.20, 0.98)	0.71 (0.30, 1.31)
$\sigma_{\mathrm{region}}$	1.05 (0.81, 1.37)	4.02 (3.46, 4.65)
σ	1.48 (1.39, 1.60)	2.11 (1.94, 2.30)

**Table 2:** Posterior estimates and 68% uncertainty intervals for SAMSAQ dimensions, considering gender, sport, and region.

	Academic motivation	Sports motivation	Career motivation
Population-level parameters			
$\beta_{0}$	3.99 (3.21, 4.74)	3.96 (3.40, 4.49)	5.07 (3.90, 6.17)
Group-level parameters			
$\sigma_{\mathrm{gender}}$	0.26 (0.06, 0.75)	0.19 (0.04, 0.68)	0.69 (0.19, 1.46)
$\sigma_{\mathrm{sport}}$	0.53 (0.25, 1.06)	0.34 (0.12, 0.80)	1.98 (1.32, 2.67)
$\sigma_{\mathrm{region}}$	2.08 (1.72, 2.50)	1.31 (1.06, 1.63)	0.83 (0.25, 1.58)
σ	0.90 (0.83, 0.98)	1.04 (0.99, 1.11)	5.16 (4.49, 5.96)

**Figure 1:**
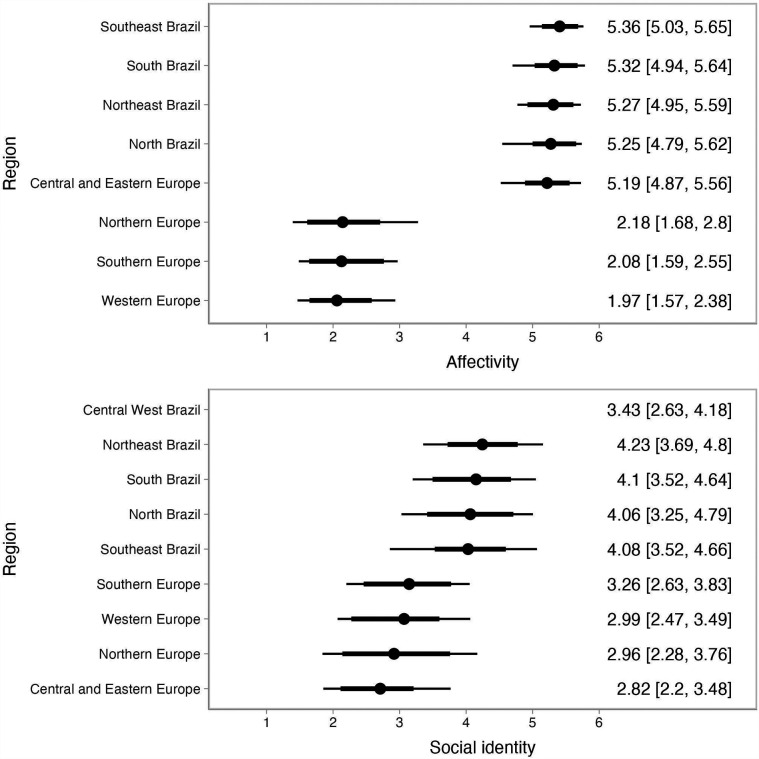
Multilevel and post-stratification results for the BIMS dimensions by region within Brazil and Europe.

**Figure 2:**
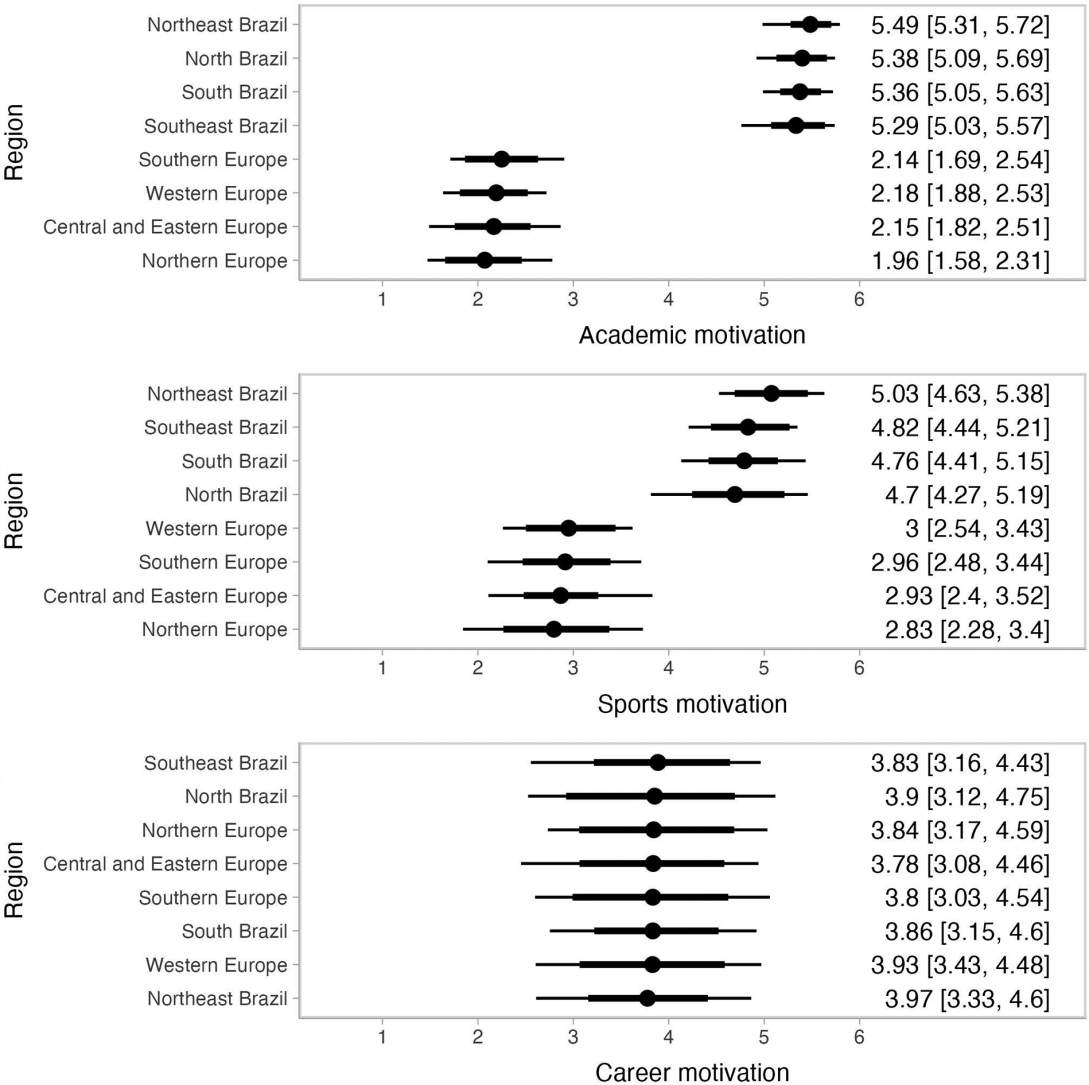
Multilevel and post-stratification results for the SAMSAQ dimensions by region within Brazil and Europe.

Figures 1, 2 illustrate the regional trends in the BIMS dimensions and the SAMSAQ dimensions, respectively, showing the variation with 68% and 90% credible intervals (representing approximately one standard deviation and a broader uncertainty range, respectively). Predictions for the Central West Brazil region are not included in the figures due to limited data and observations for post-stratification in this region. Consequently, predictions for this region primarily reflect the multilevel model estimates and associated uncertainty.

Our findings, with the exception of career motivation, indicate significant differences between Brazilian and European athletes, with Brazilian athletes reporting higher scores across all dimensions of identity and motivation. Patterns across Brazilian and European regions were consistent, showing no significant regional variation in outcomes. Additionally, there was no significant effect of gender or sport type on student-athletes’ identity or motivations toward dual careers.

## 4 Discussion

In the present study, we aimed to examine the characteristics of identity and motivation among student-athletes from Europe and Brazil from a comparative perspective. At the same time, the multiple layers that define the dual condition of students and athletes require a multidimensional approach. For this reason, it is essential to consider cultural, academic, and athletic variables in order to understand how student-athletes’ motivation and identity vary across specific social contexts.

The findings suggest that the macro-effect of the organizational and cultural context is the most significant source of influence on athletes’ motivation and identity, particularly in Brazil. Cultural nuances among European countries appear to have little impact on athletes’ responses. A similar pattern was observed in Brazil, where, despite the more developed economic status of the southern and southeastern states, no significant differences were found.

In addition to geographical, economic, and cultural differences, European states can also be categorized by the policies in place regarding dual careers, as follows: (1) State-centric regulation; (2) State as sponsor/facilitator; (3) National Sporting Federations/Institutes as intermediaries; and (4) Laissez-faire/No Formal Structures (European Commission, 2016). In the group of countries with state-centric regulation (France, Hungary, Luxembourg, Poland, Portugal, and Spain), no northern European countries are included, suggesting that legislative frameworks may overlap with cultural differences. In contrast, Brazil adopts a more liberal and laissez-faire approach to university sports, where higher education institutions set their own sport policies without interference from federal or state governments.

In the EU, direct relationships exist between national and EU bodies. At the national level, governmental departments collaborate with universities to establish regulations and allocate financial resources. However, these macro-level relationships do not appear to translate into differences in motivation and identity or, as is possible to speculate, into a common development of dual career paths among EU member states.

The globalized culture of elite sport has given rise to the phenomenon of transnational athletic migration. Different typologies of migrating elite athletes require careful consideration in research and international policies (22). Since identity is partly shaped by others’ recognition, individuals can suffer significant harm if society reflects a demeaning image of them. Misrecognition can be a form of moral harm that undermines self-esteem and the capacity for full personhood. This issue became particularly acute with modernity, where recognition could no longer be based solely on tradition or ritual. Individuals were expected to develop themselves independently, but soon realized that they relied on others to recognize their authentic selves. This may be the case for student-athletes, who face public scrutiny in the sport arena, which in the two events considered in this study, receives substantial media attention.

Student-athletes represent a unique group because their identity is formed and reinforced within two dominant social contexts: academics and athletics. This dual context can foster the belief that athletic and academic identities reflect athletes’ perceptions of themselves as a dual persona—both athlete and student. Recent studies have demonstrated a direct connection between institutional organization and athletic identity (13, 23).

In Europe, it is believed that most athletes enroll in higher education not to pursue a sporting career but to obtain an education that will lead to better job opportunities in the future, as they believe financial independence through sport is unlikely (24, 25). However, in the present study, our estimates show that Brazilian students exhibit higher levels of academic motivation, possibly due to the elite sample of European athletes.

The higher levels of academic motivation observed among Brazilian student-athletes may be attributed to several socio-economic factors. In a nation where social mobility is often linked to educational certification, pursuing higher education can be viewed as a crucial pathway to future success. Moreover, the inherent challenges of navigating a less structured university sports system in Brazil may foster a heightened sense of determination and resilience. The relative prestige of university education in Brazil, coupled with the desire to overcome economic disparities, could act as a powerful motivator. The cultural value placed on sport, and the potential for it to provide social and economic opportunities, may further amplify academic motivation. It is also important to consider that the elite athletes from Brazil, that were part of this study, have most likely had to overcome many obstacles to arrive at the level they are at, which can increase their motivation. The economic realities in Brazil, where many talented athletes face financial constraints, could also drive higher motivation, as they may view academic achievement as a safety net, ensuring a stable future beyond their athletic careers. The high levels of social inequality present in Brazil, may also influence the high level of motivation, as the athletes may view this as an upward mobility opportunity.

Although significant differences exist among states regarding national legislation and policy recommendations, student-athletes seem either unaware of these policies or perceive them as too distant to have a tangible impact on their academic and athletic careers. We assumed that student-athletes from states with centralized political decisions regarding university sports, as seen in some European countries, would exhibit higher levels of academic and sport motivation compared to other organizational models (12). However, this was not the case, and we suggest that the homogeneity of the sample, consisting of elite athletes competing at an international level, is the likely cause for this result.

Although significant differences exist among states regarding national legislation and policy recommendations, student-athletes seem either unaware of these policies or perceive them as too distant to have a tangible impact on their academic and athletic careers. We assumed that student-athletes from states with centralized political decisions regarding university sports, as seen in some European countries, would exhibit higher levels of academic and sport motivation compared to other organizational models (12). However, this was not the case, and we suggest that the varying levels of elite participation across our Brazilian and European samples, rather than simple homogeneity, is the likely cause for this result. In Brazil, a broader range of athletic levels participate, potentially diluting typical gender disparities and influencing overall motivation patterns.

The findings also indicate that Brazilian student-athletes strongly desire to develop a dual career. This contrasts with the lack of national policies addressing dual careers, even at the level of federal states. In a previous study (12), model estimations suggested that Brazilian student-athletes exhibit higher affectivity and social identity compared to a sample of Portuguese student-athletes. Given that university sports programs in Brazil tend to be less structured, it seems that Brazilian university contexts may provide more opportunities for sport participation, allowing students with varying levels of sport experience to engage in athletics while supporting elite student-athletes in national competitions. In Europe, athletes’ development is closely tied to the club system and is less school-based (26, 27), which could explain why European athletes exhibit lower levels of affectivity. The social identity values align with the diverse organizational models across continents, with student-athletes from Brazilian universities displaying higher scores than their European counterparts.

There is no ideal strategy to balance academic and athletic careers for student-athletes (24). However, our results suggest that cultural, social, and political contexts directly affect motivation to pursue a dual career. Clearly, local university policies seem to have the greatest impact on their student-athletes. Additionally, previous studies in Europe (28) and Brazil (12) found local differences in motivation and identity. In our study, these differences appear to diminish, suggesting a cultural alignment within continents that warrants further attention.

The importance of providing athletes with opportunities to develop academic careers in a high-level sport environment has been emphasized, noting that this is one of the prime factors athletes consider when choosing a university (29). On a global scale, athletes face similar challenges, but understanding how student-athletes identify with their own reality, how they face these challenges, and how they balance the demands of sport and education requires comparative studies of the contexts in which they are engaged (24, 26, 30). Thus, innovative approaches to understanding the dual nature of their training (academic and athletic) are needed. Transnational studies and projects can play a critical role in identifying best practices to reduce dropout rates in both academic and sports careers, promoting successful transitions for athletes, and advising governments, sports organizations, and educational institutions on better management of sports and education (31, 32). We acknowledge that the empirical nature of our research does not diminish the complexity of how sport perceptions vary across states.

While traditionally males are seen as more athletically oriented, our study found no gender differences. This may be due to the varying levels of elite participation across our Brazilian and European samples. In Brazil, a broader range of athletic levels participate, potentially diluting typical gender disparities. Additionally, the intense demands of elite student-athlete life, balancing academics and sports, may create a shared motivational landscape that minimizes gender differences. Future research with more diverse samples is needed to explore this further.

The limitations of the current study primarily arise from its cross-sectional and quantitative design. Further research is necessary to gain a more comprehensive understanding of the dual commitments required of student-athletes and their institutions. It is essential to move beyond the parochialism and ethnocentrism that often characterize much of the sport research literature and to examine the factors influencing motivation and identity from a global perspective. The phenomenon of university sport requires a more universal understanding, without preconceived models. While the U.S. commercial model is frequently presented as a benchmark, its applicability to other regions of the world remains limited. This study’s focus on elite athletes in specific events limits generalizability to broader student-athlete populations. Future research should prioritize diverse samples, longitudinal designs, and consider a wider range of regions to capture the complexities of student-athlete experiences. Additionally, the study’s cross-sectional design and data collection during specific events may introduce bias. Future studies should consider the time of year, proximity to events, and wider athlete skill levels to improve generalizability.

Our findings underscore the need for tailored interventions to support student-athletes in diverse contexts. For educators, this implies developing flexible academic programs that acknowledge the rigorous demands of elite sport. Policymakers should consider implementing or refining dual career policies, drawing on best practices from various models while recognizing the specific cultural and institutional landscapes. For sports organizations, fostering a supportive environment that values both athletic and academic achievements is crucial. This could involve providing mentorship programs, academic advising, and resources to navigate the dual career path. Specifically, in Brazil, given the lack of federal support, it becomes imperative that universities develop robust internal policies and support structures. Furthermore, sports organizations should advocate for increased public and private investment in dual career initiatives. It would be a major step forward if higher education institutions create dual career programs that provide academic flexibility, psychological support, and career counseling.

In this regard, we have deliberately avoided the dichotomy between cultural relativism and universalism, as it is not productive in the context of this study. Our approach to the data has been free from a priori assumptions. Furthermore, it is imperative to move beyond ethnographic approaches and adopt more sophisticated methodologies that allow for the accumulation of knowledge that can be meaningfully compared and interpreted across different contexts. In this field, the distribution of samples is rarely normal, requiring the use of advanced statistical techniques beyond traditional inferential methods. Lastly, it is crucial to conduct comparative studies at the global level to identify effective strategies. Thousands of elite athletes are currently enrolled in higher education and contribute to both national and international competitions, demonstrating their commitment and skill.

## Data Availability

The datasets presented in this study can be found in online repositories. The names of the repository/repositories and accession number(s) can be found below: https://osf.io/grb56/.
